# A retrospective epidemiological study of screening-derived solid cancer incidence proxy rates in South Korea before and after COVID-19 using Korean National Health Insurance Service data

**DOI:** 10.1371/journal.pone.0360040

**Published:** 2026-10-16

**Authors:** Hyeran Jung, Minsun Jung

**Affiliations:** Department of Pathology, Yonsei University College of Medicine, Seoul, Republic of Korea; Athens Medical Group, Psychiko Clinic, GREECE

## Abstract

**Background:**

The COVID-19 pandemic disrupted cancer screening and diagnostic pathways globally. Aggregate screening-derived indicators require cautious interpretation because they do not represent confirmed cancer registry incidence but might reflect program participation, referral patterns, and denominator composition.

**Objective:**

To evaluate pre- and post-COVID-19 changes in selected solid cancer screening-derived incidence proxy rates in South Korea, and to estimate model-based proxy rates through 2028.

**Methods:**

This retrospective ecological longitudinal study analyzed publicly available aggregate annual cancer-screening statistics from the Korean National Health Insurance Service (NHIS) and Korean Statistical Information Service (KOSIS) for six solid cancer programs (stomach, liver, colorectal, breast, cervical, and lung cancer). Annual proxy rates per 100,000 screened or eligible persons were calculated. Pre-COVID (2010–2019) and post-COVID (2020–2024) means were compared using Welch t-tests with Mann–Whitney U sensitivity analyses. Ordinary least-squares (OLS) forecasts were generated for 2025–2028. Interrupted time-series analysis was not feasible owing to an insufficient post-COVID observation window.

**Results:**

Post-COVID changes in proxy rates were heterogeneous across selected programs. Breast cancer proxy rates increased by 17.1% (Welch p = 0.002) and liver cancer proxy rates by 83.6% (Welch p = 0.002); the liver cancer increase reflects denominator restriction to a high-risk population rather than a true epidemiological surge. Stomach cancer decreased by 23.3% (Welch p = 0.001), cervical cancer by 56.9% (Welch p = 0.005), and colorectal cancer by 25.8% (Welch p = 0.007). Lung cancer data were available only from 2019 to 2024 (program inception) and were interpreted descriptively.

**Conclusions:**

Aggregate South Korean national screening data show pandemic-era temporal associations with heterogeneous changes in selected solid cancer program proxy rates. These findings support an indirect screening-disruption and ascertainment interpretation rather than causal proof of a direct COVID-19 effect on solid cancer incidence.

## Introduction

The COVID-19 pandemic disrupted the delivery of preventive health services, diagnostic procedures, outpatient visits, and hospital-based follow-up worldwide. Cancer screening programs were particularly vulnerable because many examinations and confirmatory diagnostic procedures require scheduled visits and adequate health-system capacity [1–3].

Screening-derived cancer indicators are useful population-level signals, but they are not identical to patient-level cancer registry incidence [2,3]. The numerator in national screening program statistics captures positive or referred screening findings, not pathologically confirmed diagnoses. The denominator represents the number of persons screened or eligible under the program. These rates are therefore correctly described as “screening-derived proxy rates.” They may be influenced by participation in screening, referral patterns, diagnostic access, delayed work-up, catch-up screening, denominator composition changes, and program eligibility criteria. Pandemic-era comparisons must be framed as temporal epidemiological signals rather than direct causal evidence of a COVID-19 effect on cancer biology [1–3].

Unlike the United States and most European countries, South Korea maintains a national cancer screening program covering stomach cancer and hepatocellular carcinoma (liver cancer) in addition to colorectal, breast, and cervical cancer, owing to their elevated incidence in the South Korean population [4,5]. The liver cancer program targets a restricted high-risk denominator, specifically persons with liver cirrhosis or chronic hepatitis B or C virus infection, rather than the general age-eligible population. A national high-risk lung cancer screening program was introduced in 2019. These distinctive program structures affect proxy rate interpretation and must be considered when comparing rates across cancer types or with international data.

This retrospective study provides a rigorous, solid-cancer-focused analysis of aggregate South Korean screening-derived proxy rates before and after the COVID-19 pandemic, following the Strengthening the Reporting of Observational Studies in Epidemiology (STROBE) reporting guidance [6] and ICMJE recommendations for scholarly publication [7]. Data were obtained from the NHIS and KOSIS [5].

## Materials and methods

### Study design and data sources

This retrospective ecological longitudinal analysis used publicly available, fully deidentified aggregate South Korean national cancer-screening statistics. Data were sourced from the NHIS and KOSIS [4,5]; all data are available as annual aggregate summary tables from the KOSIS portal (https://kosis.kr). The analytic dataset covered stomach, liver, colorectal, breast, cervical, and lung cancer screening programs. No individual-level NHIS records, linked cancer-registry records, or personally identifiable information were used.

This study was granted exemption from full Institutional Review Board review (IRB No. 202511-SB-218-01, confirmed 14 November 2025) by the Chungnam National University Institutional Review Board (CNUIRB), in accordance with applicable national regulations governing secondary use of publicly available aggregate data. Chungnam National University served as the institutional sponsor for the IRB application; the principal investigator (H.J.) is affiliated with Yonsei University College of Medicine. The date of data access was recorded as 17 November 2025.

### Outcomes and period definition

The primary outcome was the annual screening-derived solid cancer incidence proxy rate per 100,000 screened or eligible persons. The numerator is defined as the number of persons identified with a suspected or screen-positive finding meeting program-specific referral criteria in a given year; it does not represent pathologically confirmed cancer diagnoses. The denominator is the number of persons who underwent the screening examination or, where reported, the number eligible under program criteria. Definitions for each program are provided in Table 1.

**Table 1:** Solid cancer screening program data sources and analytic use, South Korea.

Cancer program	Years	Numerator definition	Denominator	Analytic use
**Stomach cancer**	2010–2024	Screen-positive findings (endoscopy or UGI) meeting referral criteria	Persons who underwent stomach cancer screening in the program year	Pre-/post-COVID comparison; 5-program composite
**Liver cancer**	2010–2024	Suspected hepatocellular carcinoma identified by ultrasonography/AFP	High-risk eligible persons (liver cirrhosis or chronic hepatitis B/C) enrolled in liver cancer screening	Pre-/post-COVID comparison; denominator restriction note applies
**Colorectal cancer**	2010–2024	Screen-positive findings (FOBT or colonoscopy) meeting referral criteria	Persons who underwent colorectal cancer screening in the program year	Pre-/post-COVID comparison (2 years missing); 5-program composite
**Breast cancer**	2010–2024	Suspected findings on mammography meeting recall/referral criteria	Women aged ≥40 years who underwent breast cancer screening	Pre-/post-COVID comparison; 5-program composite
**Cervical cancer**	2010–2024	Suspected findings on Pap smear meeting referral criteria	Women aged ≥20 years who underwent cervical cancer screening	Pre-/post-COVID comparison; 5-program composite
**Lung cancer**	2019–2024	Suspected findings on low-dose CT meeting referral criteria	High-risk eligible persons (age 54–74, heavy smoking history) enrolled in lung cancer screening	Descriptive only; program commenced 2019; short pre-COVID window

AFP, alpha-fetoprotein; CT, computed tomography; FOBT, fecal occult blood test; UGI, upper gastrointestinal series.

The pre-COVID-19 period was defined as 2010–2019 (where data were available); the post-COVID-19 period was defined as 2020–2024. Lung cancer screening data were available only from 2019 to 2024 because the South Korean national high-risk lung cancer screening program commenced in 2019. Lung cancer was therefore interpreted descriptively and excluded from the stable 5-program composite analysis.

### Statistical analysis

Annual proxy rates were calculated as detected cases divided by screened or eligible persons, multiplied by 100,000. Linear trend models estimated annual slopes and Pearson correlation coefficients. Pre-/post-COVID-19 mean proxy rates were compared using Welch two-sample t-tests; Mann–Whitney U tests were performed as non-parametric sensitivity analyses. Given the exploratory nature of this analysis, multiple comparisons were not adjusted; all results should be interpreted as hypothesis-generating.

Interrupted time-series (ITS) analysis was considered but not performed, because only five post-COVID annual observations (2020–2024) were available for most programs, which is insufficient for reliable ITS coefficient and confidence-interval estimation. Accordingly, ITS is not described further in the Methods or Results.

Forecasts for 2025–2028 were generated using OLS regression with centered calendar year. Where sufficient observations permitted, a binary post-2020 indicator was included. Uncertainty intervals are 95% prediction intervals (PI), reflecting out-of-sample forecast uncertainty, and are not confidence intervals. Negative lower PI bounds were truncated to zero for display only, as proxy rates cannot be negative. Forecast point estimates are presented to one decimal place; higher numerical precision would not be scientifically warranted given the short time series.

### Ethics statement

This study was granted exemption from full Institutional Review Board review (IRB No. 202511-SB-218-01, confirmed 14 November 2025) by the CNUIRB, in accordance with applicable South Korean national regulations for secondary analysis of publicly available aggregate administrative data. The requirement for individual informed consent was not applicable because no individual-level data, identifiable personal information, or direct participant contact was involved. All researchers complied with CNUIRB conditions: research was conducted according to the approved study plan and records will be retained for at least three years following study completion.

## Results

### Analytic dataset

Five programs provided data spanning 2010–2024 (stomach, liver, colorectal, breast, and cervical cancer) and were included in the primary pre-/post-COVID-19 comparisons. The lung cancer program, which commenced in 2019, was analyzed descriptively only. Colorectal cancer had two pre-COVID years of missing program data (2013 and 2016), stomach cancer had one pre-COVID year missing (2013), and cervical cancer had one pre-COVID year missing (2014). Colorectal cancer had two pre-COVID years of missing program data (2013 and 2016), stomach cancer had one pre-COVID year missing (2013), and cervical cancer had one pre-COVID year missing (2014). Full program details are provided in Table 1.

### Pre-/post-COVID-19 proxy rate comparisons

Post-COVID-19 changes in screening-derived proxy rates were heterogeneous across selected solid cancer programs (Table 2 and Figs 1 and 2). Breast cancer proxy rates increased by 17.1% (pre-COVID mean 146.8, post-COVID mean 171.9 per 100,000; Welch p = 0.002; Mann–Whitney p = 0.019). Liver cancer proxy rates showed the largest proportional change (+83.6%; Welch p = 0.002); however, this increase most plausibly reflects changes in the restricted high-risk denominator (persons with liver cirrhosis or chronic hepatitis B/C) rather than a true epidemiological surge in hepatocellular carcinoma incidence.

**Table 2:** Pre-/post-COVID-19 comparison of screening-derived solid cancer proxy rates in South Korea (2010–2024).

Cancer or composite	n (pre/post)	Pre-COVID mean*	Post-COVID mean*	Absolute change	Relative change (%)	Welch p	MW p
**Breast cancer**	10/ 5	146.8	171.9	+25.1	+17.1	0.002	0.019
**Cervical cancer**	9/ 5	17.6	7.6	−10.0	−56.9	0.005	—
**Colorectal cancer†**	8/ 5	64.2	47.6	−16.6	−25.8	0.007	0.011
**Liver cancer‡**	10/ 5	860.8	1580.5	+719.6	+83.6	0.002	0.055
**Lung cancer§**	1/ 5	—	3664.4	—	−20.0§	NA	NA
**Stomach cancer†**	9/ 5	138.9	106.5	−32.4	−23.3	0.001	0.002
**5-Program composite (excl. lung)**	7/ 5	110.5	107.0	−3.5	−3.2	0.256	0.440
**Composite incl. lung (2019 onward)**	1/ 5	111.9	125.9	+14.0	+12.5	—	—

* Proxy rates per 100,000 screened or eligible persons; ‘Pre-COVID mean’ and ‘Post-COVID mean’ are mean annual proxy rates within the respective period. Pre-COVID period: 2010–2019; post-COVID period: 2020–2024. n (pre/post) = number of annual observations in each period included in analysis. MW, Mann–Whitney U test; NA, insufficient annual observations for the test. † Colorectal cancer had 2 pre-COVID years missing (2013, 2016); stomach cancer had 1 pre-COVID year missing (2013); cervical cancer had 1 pre-COVID year missing (2014). ‡ Liver cancer proxy rates are not comparable with other programs on a per-100,000 basis because the denominator is restricted to a high-risk subpopulation (liver cirrhosis or chronic hepatitis B/C) rather than the general age-eligible population; see Discussion. § Lung cancer pre-COVID period has only one observation (2019); formal pre-/post-mean comparison is not possible. Relative change is approximate (2019 rate vs. 2020–2024 mean). ¶ The 5-program composite (excluding lung) is the unweighted arithmetic mean of annual proxy rates across the five stable programs (stomach, liver, colorectal, breast, cervical), computed only for calendar years with complete data across all five programs; n = 7 pre-COVID and 5 post-COVID reflects the years retained after excluding missing-data years. Specifically, the following years were excluded from the pre-COVID composite because at least one of the five programs lacked data: 2013 (colorectal and stomach cancer data missing), 2014 (cervical cancer data missing), and 2016 (colorectal cancer data missing). No post-COVID years were missing across any of the five programs. The composite therefore uses 7 pre-COVID years (2010, 2011, 2012, 2015, 2017, 2018, 2019) and 5 post-COVID years (2020–2024). The 6-program composite (including lung) is computed identically but restricted to 2019–2024; the post-COVID composite mean of 125.9 reflects the unweighted mean of all six programs’ annual proxy rates for 2020–2024. Because the pre-COVID window for the 6-program composite contains only one observation (2019; n = 1), inferential Welch t-test and Mann–Whitney statistics cannot be reliably estimated and are therefore not reported (shown as —).

**Fig 1:**
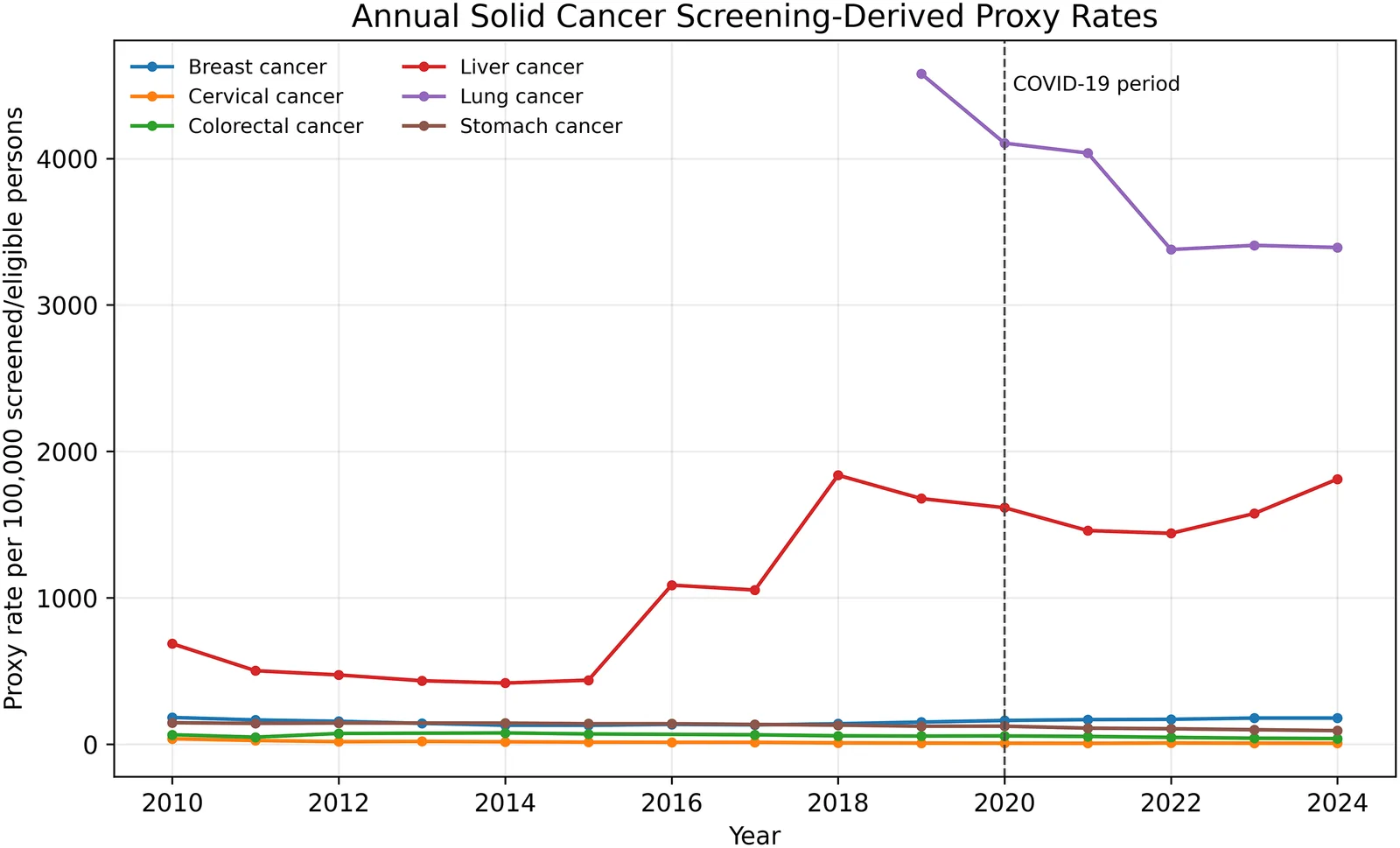
Annual solid cancer screening-derived proxy rates per 100,000 screened or eligible persons, South Korea, 2010–2024. The vertical dashed line indicates 2020, the start of the COVID-19 pandemic period. Note that liver cancer and lung cancer proxy rates are plotted on a shared y-axis with other programs; cross-program rate comparisons are not meaningful because denominators differ by program (see Table 1).

**Fig 2:**
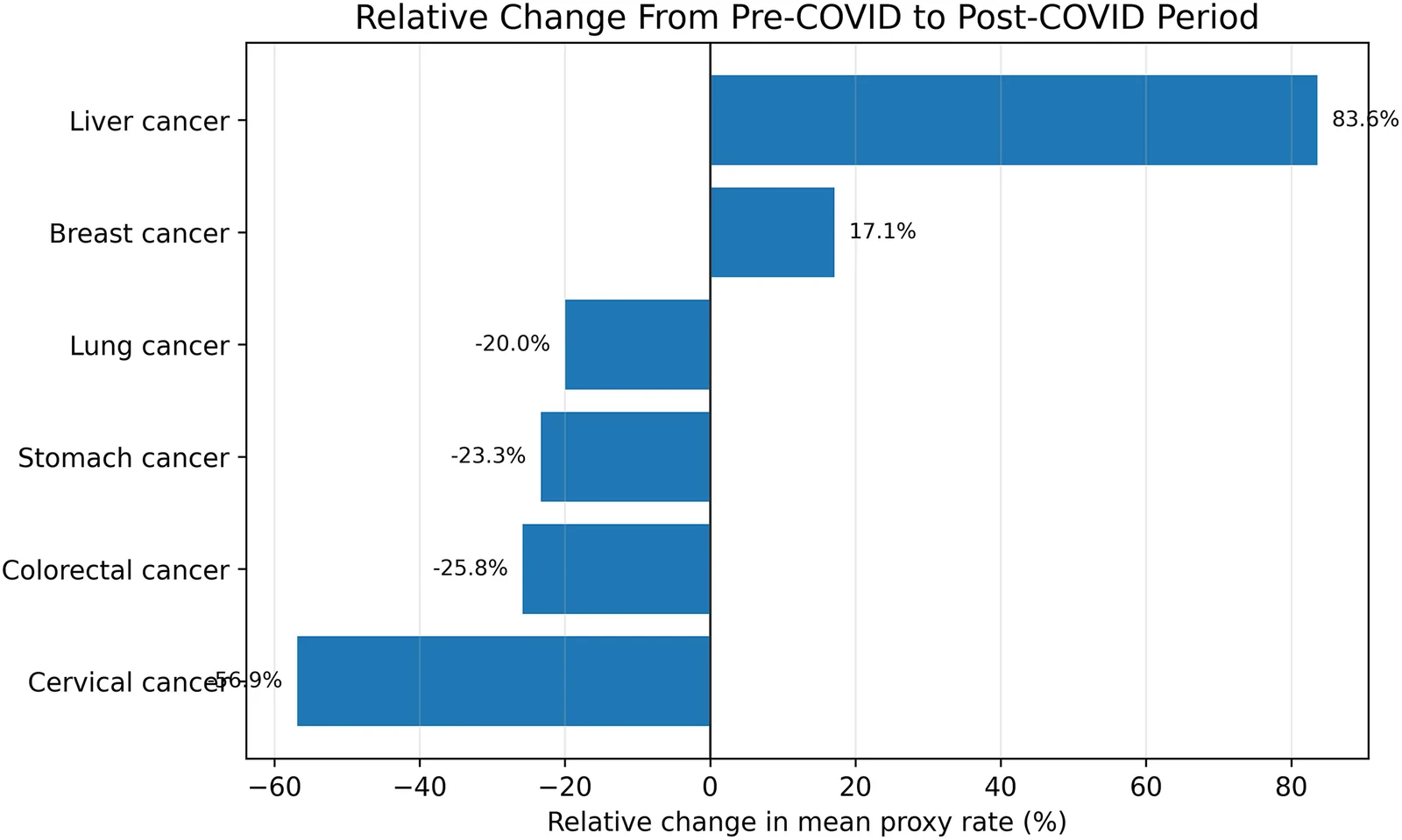
Relative change (%) in screening-derived solid cancer proxy rates from the pre-COVID (2010–2019) to post-COVID (2020–2024) period. Lung cancer is excluded from this figure because only one pre-COVID observation (2019) is available, precluding a valid pre-/post-COVID-19 comparison. Cervical cancer label is repositioned to prevent overlap. Cross-program magnitude comparisons should be made with caution given differing denominators.

Stomach cancer proxy rates decreased by 23.3% (Welch p = 0.001), cervical cancer by 56.9% (Welch p = 0.005), and colorectal cancer by 25.8% (Welch p = 0.007). The stable 5-program composite (excluding lung) changed by −3.2% (Welch p = 0.256), a modest, non-significant decrease. The 6-program composite including lung (restricted to 2019–2024) showed a post-COVID mean of 125.9 compared with a single pre-COVID observation of 111.9 (2019); because only one pre-COVID annual value is available, inferential statistics cannot be reliably calculated and a descriptive comparison is presented instead. The apparent increase in the 6-program composite reflects the dominant weight of large-scale lung cancer proxy rates rather than a concordant increase across all programs. These two composites measure different quantities and should not be compared directly.

For the Mann–Whitney sensitivity analysis, liver cancer diverged from the Welch result (Welch p = 0.002 vs. Mann–Whitney p = 0.055). This divergence reflects the rank-based sensitivity of the Mann–Whitney test to the small post-COVID sample (n = 5 years) relative to pre-COVID (n = 10 years), combined with the large absolute difference in mean rates. The Mann–Whitney result is more conservative and should be considered the primary non-parametric estimate for liver cancer.

### Forecasts to 2028

OLS-based forecasts for 2025–2028 are presented in Table 3 and Figs 3 and 4. Forecasts for liver cancer and lung cancer carry the widest prediction intervals, reflecting high within-program variability and a very short observation window, respectively. Liver cancer and lung cancer forecast lines are omitted from Fig 3 owing to scale differences and forecast instability; corresponding estimates are tabulated in Table 3.

**Table 3:** OLS model-based forecasts to 2028 for solid cancer screening-derived proxy rates in South Korea.

Cancer or composite	2028 predicted rate*	Lower 95% PI	Upper 95% PI	Estimated cases†	Model
**Breast cancer**	155.0	117.4	192.5	6,925	OLS: year + post-2020
**Cervical cancer**	0.0	0.0	4.8	0.0	OLS: year + post-2020
**Colorectal cancer**	42.0	16.4	67.7	2,649	OLS: year + post-2020
**Liver cancer‡**	2364.0	1537.2	3190.8	11,524	OLS: year + post-2020
**Lung cancer§**	2204.2	1105.5	3302.8	3,780	OLS: centered year
**Stomach cancer**	90.1	74.5	105.6	7,713	OLS: year + post-2020
**5-Program composite (excl. lung)**	100.8	82.5	119.0	25,615	OLS: year + post-2020
**Composite incl. lung**	124.8	102.2	147.3	31,937	OLS: year + post-2020

OLS, ordinary least squares; PI, prediction interval (95%); these are out-of-sample prediction intervals, not confidence intervals. * Per 100,000 screened or eligible persons; point estimates are rounded to one decimal place. † Estimated cases are calculated using the most recent available screened or eligible population volume for planning purposes only; they are not observed NHIS outcomes. ‡ Liver cancer forecasts carry additional uncertainty owing to high within-program rate variability and denominator instability. § Lung cancer forecast is based on only six annual observations (2019–2024); uncertainty is very high.

**Fig 3:**
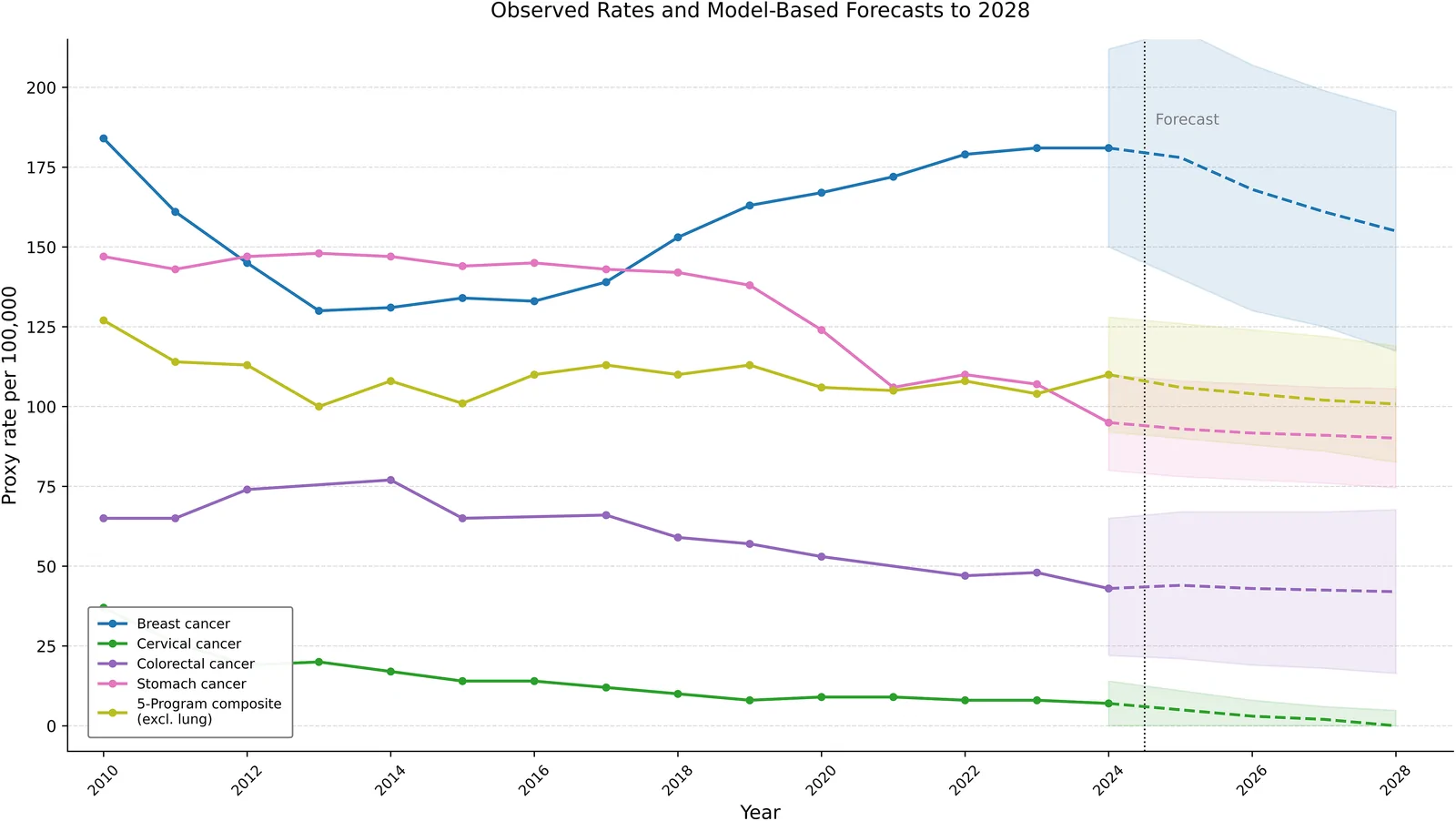
Observed proxy rates and model-based OLS forecasts to 2028 for stomach, colorectal, breast, and cervical cancer, and the 5-program composite (excluding lung), per 100,000 screened or eligible persons. Dashed forecast lines use the same color as the corresponding observed-period trend line. Uncertainty ranges for all programs are tabulated in Table 3. Liver cancer and lung cancer forecast lines are omitted owing to scale differences and forecast instability; see Table 3.

**Fig 4:**
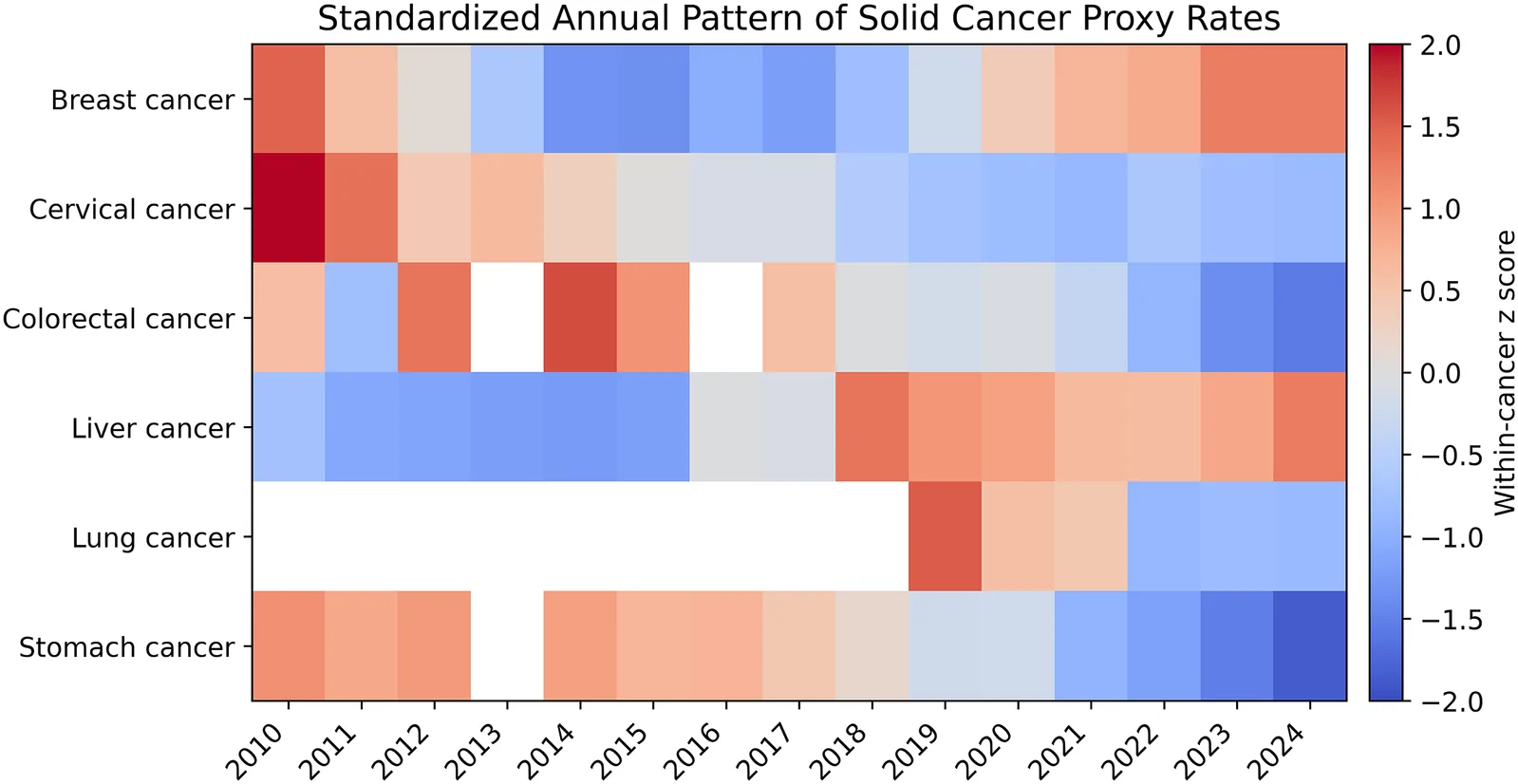
Heatmap of annual solid cancer screening-derived proxy rates standardized within each cancer program (within-cancer z-score), South Korea, 2010–2024. Red cells indicate above-mean rates; blue cells indicate below-mean rates. Lung cancer program data begin in 2019; cells for 2010–2018 are blank.

## Discussion

This solid-cancer screening analysis found no uniform pandemic-associated change across all selected South Korean national cancer-screening programs. Instead, results show a heterogeneous pattern in which some screening-derived proxy rates increased and others decreased after 2020, consistent with the complex interplay of screening disruption, catch-up effects, denominator changes, and ascertainment shifts during and after the COVID-19 pandemic (Table 2 and Figs 1 and 2).

The most plausible overall interpretation is that the post-COVID-19 period was temporally associated with changes in screening participation, diagnostic access, and ascertainment rather than direct biological causation [1–3]. Higher breast cancer proxy rates likely reflect a combination of catch-up screening after pandemic-related delays, differential participation patterns, or increased detection sensitivity in the post-pandemic period.

The 83.6% increase in liver cancer proxy rates warrants specific contextual explanation. Published South Korean epidemiological data indicate that age-standardized liver cancer incidence has declined since approximately 2008–2010 [8], rendering a true epidemiological increase of 83.6% highly implausible. The South Korean liver cancer screening program targets a restricted high-risk denominator: only persons with confirmed liver cirrhosis or chronic hepatitis B or C virus infection are eligible [4,5]. Any change in the composition or size of this high-risk registry—such as shifts in hepatitis B treatment coverage, changes in cirrhosis ascertainment, or updates to eligibility criteria—would directly and substantially alter the proxy rate without a corresponding change in hepatocellular carcinoma incidence. The liver cancer proxy rates observed in this analysis (860–1,580 per 100,000 high-risk eligible persons) are therefore not comparable to proxy rates from programs using a general age-eligible denominator, and should not be interpreted as evidence of a biological cancer surge.

Lower stomach, colorectal, cervical, and lung proxy rates are consistent with patterns reported in other high-income countries during the COVID-19 pandemic. Dinmohamed et al. reported substantially fewer cancer diagnoses in the Netherlands during the acute COVID-19 period [3], and Maringe et al. modelled significant excess cancer deaths attributable to diagnostic delays in England [2]. These international findings support the hypothesis that reduced screening participation, delayed follow-up, and disrupted diagnostic pathways during 2020–2021 contributed to lower detection rates—not necessarily lower underlying cancer occurrence. The direction and magnitude of changes in South Korea may differ from Western countries given differences in national screening program organization, epidemic timing, and governmental response.

Forecasts through 2028 suggest heterogeneous recovery trajectories rather than uniform rebound (Table 3 and Figs 3 and 4). Cervical cancer proxy rates are projected to approach zero by 2028, which may reflect the sustained impact of high HPV vaccination coverage among cohorts now entering the screening-age range. These forecasts are model-based planning estimates derived from short annual time series and should therefore be validated against future observed NHIS data [9].

The ecological design with aggregate data cannot formally partition the relative contributions of COVID-19 biological effects, individual-level healthcare-seeking behavior changes, screening program policy modifications, or denominator composition changes. Screening volume, participation rates, diagnostic referral completion rates, and denominator composition were not directly measurable from the available aggregate KOSIS/NHIS statistics.

## Limitations

First, the analytic dataset consists of aggregate screening program statistics without individual-level NHIS records, cancer-registry records, COVID-19 infection or vaccination history, or patient-level covariates. Individual-level causal inference is not supported.

Second, screening-derived proxy rates are not equivalent to cancer incidence. The numerator captures screen-positive findings meeting program referral criteria; it does not represent pathologically confirmed diagnoses. Rates are influenced by participation, referral, diagnostic work-up completion, follow-up adherence, and screening eligibility criteria.

Third, the ecological design cannot establish that COVID-19 directly caused or prevented cancer. Results support temporal association and indirect health-system disruption [1–3]; causal claims are not warranted.

Fourth, lung cancer data were available only for 2019–2024 owing to the 2019 introduction of the national high-risk lung cancer screening program, precluding stable pre-pandemic comparison and reliable forecasting.

Fifth, ITS analysis was not performed because only five post-COVID annual observations were available, which is insufficient for reliable ITS coefficient estimation.

Sixth, multiple statistical tests were performed across six programs and two composite measures without adjustment for multiple comparisons. All p-values should be interpreted in an exploratory framework.

Seventh, forecasts are model-based planning estimates from short annual time series. Numerical precision of forecast point estimates does not imply predictive accuracy; prediction intervals are wide and likely underestimate true out-of-sample uncertainty.

## Conclusions

In aggregate South Korean national cancer-screening data, the post-COVID-19 period was temporally associated with heterogeneous changes in selected solid cancer program proxy rates. The findings support an indirect screening-disruption and ascertainment interpretation rather than causal proof that COVID-19 directly caused or prevented solid cancer incidence. The anomalously high liver cancer proxy rates most plausibly reflect the restricted high-risk denominator of the South Korean liver cancer screening program, not a biological surge in hepatocellular carcinoma incidence. Ongoing surveillance of screening participation rates and cancer diagnostic pathways is warranted to monitor the long-term consequences of pandemic-related healthcare disruption in South Korea and to inform recovery strategies.

## Supporting information

S1 File Graphical abstract. (TIF)
